# Identification and Functional Analysis of *ThADH1* and *ThADH4* Genes Involved in Tolerance to Waterlogging Stress in *Taxodium* hybrid ‘Zhongshanshan 406’

**DOI:** 10.3390/genes12020225

**Published:** 2021-02-04

**Authors:** Lei Xuan, Jianfeng Hua, Fan Zhang, Zhiquan Wang, Xiaoxiao Pei, Ying Yang, Yunlong Yin, David L. Creech

**Affiliations:** 1 Jiangsu Province Engineering Research Center of *Taxodium* Rich. Germplasm Innovation and Propagation, Institute of Botany, Jiangsu Province and Chinese Academy of Sciences (Nanjing Botanical Garden Mem, Sun Yat-Sen), Nanjing 210037, China; 13851991791@163.com (L.X.); mumizhongfeng@126.com (F.Z.); zhiquanjiejie@163.com (Z.W.); x18944051197@126.com (X.P.); yingyang@cnbg.net (Y.Y.); yinyl066@sina.com (Y.Y.); 2 Arthur Temple College of Forestry and Agriculture, Stephen F. Austin State University, Nacogdoches, TX 75962, USA; dcreech@sfasu.edu

**Keywords:** *Taxodium* hybrid ‘Zhongshanshan 406’, ThADH, waterlogging, expression patterns

## Abstract

The *Taxodium* hybrid ‘Zhongshanshan 406’ (*T*. hybrid ‘Zhongshanshan 406’) [*Taxodium mucronatum* Tenore × *Taxodium distichum* (L.). Rich] has an outstanding advantage in flooding tolerance and thus has been widely used in wetland afforestation in China. *Alcohol dehydrogenase genes* (*ADHs*) played key roles in ethanol metabolism to maintain energy supply for plants in low-oxygen conditions. Two *ADH* genes were isolated and characterized—*ThADH1* and *ThADH4* (GenBank ID: AWL83216 and AWL83217—basing on the transcriptome data of *T*. hybrid ‘Zhongshanshan 406’ grown under waterlogging stress. Then the functions of these two genes were investigated through transient expression and overexpression. The results showed that the ThADH1 and ThADH4 proteins both fall under ADH III subfamily. ThADH1 was localized in the cytoplasm and nucleus, whereas ThADH4 was only localized in the cytoplasm. The expression of the two genes was stimulated by waterlogging and the expression level in roots was significantly higher than those in stems and leaves. The respective overexpression of *ThADH1* and *ThADH4* in *Populus* caused the opposite phenotype, while waterlogging tolerance of the two transgenic *Populus* significantly improved. Collectively, these results indicated that genes *ThADH1* and *ThADH4* were involved in the tolerance and adaptation to anaerobic conditions in *T*. hybrid ‘Zhongshanshan 406’.

## 1. Introduction

In China, there are approximately 53.6 million hectares of wetlands. However, in recent decades, as a result of human activities, the ecological function of much of the wetlands has seriously deteriorated. Considering the Yangtze River Basin as an example, under the influence of urbanization and industrialization, nearly 60% of the wetlands in the middle and lower reaches of the Yangtze River are at risk of gradually disappearing, with the wetland ecosystem becoming seriously damaged [1]. The selection and cultivation of plants, especially flooding-tolerant trees, is particularly critical for the restoration and reconstruction of wetland ecosystems in China. It is reported that, compared with herbs, few woody plants can survive prolonged flooding, especially complete submergence [2,3]. *Taxodium* hybrid ‘Zhongshanshan 406’ (*T*. hybrid ‘Zhongshanshan 406’) is an interspecific hybrid clone, produced from two *Taxodium* species, namely *Taxodium mucronatum* and *Taxodium distichum* [4], which showed a marked ability to withstand flooding. *T*. hybrid ‘Zhongshanshan 406’ has been widely planted in wetlands and lakes in China (including Poyang Lake in Jiangxi Province, Chao Lake in Anhui Province, Bagua Islet in Jiangsu Province) [5,6]. The experimental planting of *T*. hybrid ‘Zhongshanshan 406’ in the Three Gorges Reservoir of China showed an approximately 90% survival rate after the entire trees had been submerged for up to 122 days [5]. Recently, analysis of the flooding tolerance mechanism of *T*. hybrid ‘Zhongshanshan 406’ has become a research focus [7].

The ability of plants to withstand flooding and the recovery of growth after flooding depend on its carbohydrate reserves. After a long period of flooding stress, plants are finally in the state of energy starvation and the flood tolerant plants can maintain energy reserve by changing biochemical metabolic pathways [2]. In the flooding environment, the energy to maintain plant vitality mainly relies on the ethanol metabolic pathway in glycolysis to degrade glucose and glycogen, accompanied by ATP generation. Alcohol dehydrogenase (ADH) is the key enzyme in the ethanol fermentation pathway and subsequently in the adaptive anaerobic metabolism of plant tissue [8]. It has been reported that the flooding tolerance of plants was proportional to the change in ADH activity in response to flooding. *ADH* and *ADH*-like genes have been cloned from *Arabidopsis thaliana*, *Zea mays*, *Cucumis sativus* and *Vitis vinifera* [9,10,11,12,13]. *ADH* genes have been regarded as important candidates for genetic manipulation to achieve flooding tolerance in plants, through improving the adaptability to hypoxia [14,15], although there have been relatively few studies on the response of *ADH* genes to flooding in woody plants, such as *T*. hybrid ‘Zhongshanshan 406’.

In the current study, we intend to identify and characterize the *ThADH*s of *T*. hybrid ‘Zhongshanshan 406’ and to study the expression and regulation of *ThADH*s in response to waterlogging stress, using real-time quantitative polymerase chain reaction (qPCR), to identify subcellular localization of the two alcohol dehydrogenases and to introduce the *ThADH*s into flooding-sensitive *Populus* by genetic transformation. The ultimate goal of our research is to reveal the molecular mechanism of waterlogging tolerance in *T*. hybrid ‘Zhongshanshan 406’.

## 2. Materials and Methods

### 2.1. Plant Materials and Waterlogging Treatment

*T*. hybrid ‘Zhongshanshan 406’, which were planted, one per plant, in plastic pots containing 3:1:1 (*v*/*v*/*v*) clay, vermiculite and perlite, were obtained from the Institute of Botany, Jiangsu Province and the Chinese Academy of Science, Nanjing, China (35°50′ N, 45°70′ E). The plants were irrigated fully every three days. Two-year-old *T*. hybrid ‘Zhongshanshan 406’ with healthy and consistent growth were selected for stress treatment.

In early July, nine *T*. hybrid ‘Zhongshanshan 406’ plants of the similar size and development stage were selected for the experiment. The plant average height was 130 ± 5 cm and the basal diameter was 9.6 ± 0.15 mm. Normal irrigation (CK, Supplementary PIC 1), half-flooding (HF, Supplementary PIC 2) and total-submergence (TS, Supplementary PIC 3) were set up as the three treatments. The CK plants were watered normally and placed outside but adjacent to the tanks. The HF plants were flooded with a water level depth as 20 cm above the soil surface. The TS plants were completely submerged in the water. Five replicate plants were used for each treatment. On the 0, 10th, 20th, 30th, 40th, 50th and 60th days of the treatments, the leaf, stem and root tissues of CK, HF and TS plants were sampled and frozen in liquid nitrogen immediately and stored at −80 °C prior for RNA and DNA extraction.

The elite clone of hybrid poplars (*Populus davidiana* × *P. bolleana*), which were used for transient expression and genetic transformation studies, were cultivated on MS (Murashige and Skoog medium) agar medium supplemented with 0.3% (*w*/*v*) Gelrite and 3.0% (*w*/*v*) sucrose in a growth chamber at 25 °C with a 16/8 h photoperiod. Protoplasts were isolated from the leaves of 42-day-old *Populus* and genetic transformation was carried out with 28-day-old *Populus*.

### 2.2. Full-Length cDNA Cloning

We extracted the total RNA from the different organs using the RNeasy^®^ Plant Mini Kit (Qiagen, Dusseldorf, Germany). DNA contaminants were removed by DNase I (Takara, Dalian, China). Two *ThADH* genes sequence fragments were obtained from the transcriptome data from *T*. hybrid ‘Zhongshanshan 406’ plants exposed to waterlogging stress [7] and all primers were designed using the Oligo7 primer designer (http://www.mbinsights.com/). Then, 3′RACE (rapid amplification of cDNA ends) PCR and 5′RACE PCR were performed according to the manufacturer’s instructions (Takara, Dalian, China). The PCR fragments were ligated into the pMD T-19vector (Takara, Dalian, China) for sequencing. The sequences of the primers are shown in Table 1.

### 2.3. Bioinformatics and Statistical Analyses

The online BLAST software was used to analyze the DNA and protein sequences (https://blast.ncbi.nlm.nih.gov/Blast.cgi). The composition, physical and chemical characterization of the proteins were analyzed using the Expert Protein Analysis System (http://web.expasy.org/protparam/). Secondary structures of deduced amino acid sequences were predicted by the GOR IV secondary structure prediction method (https://npsa-prabi.ibcp.fr/cgi-bin/npsa_automat.pl?page=npsa_gor4.html). Protein subcellular localization prediction were performed using WoLF PSORT (https://www.genscript.com/psort/wolf_psort.html). Typical domain analysis was performed using the Pfam database (http://pfam.sanger.ac.uk/). Full-length protein sequences of various species were obtained in the non-redundant Nr protein sequence repository of the public GenBank database, performed using BLAST. Multiple alignments of the two ADH proteins were performed with ClustalX (http://www.clustal.org/clustal2/), using the default settings. Subsequently, a phylogenetic tree was constructed using the maximum likelihood method (1000 bootstraps), using MEGA7 software (http://www.megasoftware.net/) [16].

### 2.4. Quantitative Real-Time PCR Analysis

To determine the expression profiles of *ThADH1* and *ThADH4*, qPCR was performed on the Analitik Jena qTOWER2.2 PCR System (Biometra, Jena, Germany). The primers of the two *ThADHs* for qPCR were designed, based on their cDNA sequences. The *adenine phosphoribosyltransferase* (*APRT*, GenBank accession No. KX431853) gene was selected as the internal reference gene [17] (Table 1). Every sample was carried out in three biological independent replicates. The expression results were displayed in the form of the relative value 2^−ΔΔ*C*t^, where Δ*C*t represents the *C*t value of the gene subtracted from that of the reference gene [18]. All data with three biological replicates were determined using one-way analysis of variance (ANOVA) and significant differences were calculated using the Duncan’s multiple range tests (defined as *p* < 0.05) by SPSS25.0 statistical analysis software.

### 2.5. Vector Construction

The open reading frames (ORFs) of the *ThADHs* were amplified and subsequently ligated into the PCR8/GW/TOPO entry vector and then cloned into transient expression vector p2GWF7.0 and overexpression vector PH35GS, using the Gateway System (Invitrogen, Carlsbad, CA, USA). The genes were inserted downstream of the constitutive plant promoter CaMV35S in both vectors. The constructed vectors, 35S::ThADHs-GFP were introduced into *Populus* protoplasts for subcellular localizations of the ThADH proteins. The constructed vectors, 35S::ThADHs were transformed into *Agrobacterium tumefaciens* strain GV3101 for *Populus* transformation.

### 2.6. Protoplast Transfection

Hybrid poplar tissue culture seedlings (42-days-old) was grown in tissue culture and protoplasts were released, using cellulase and pectate lyase [19]. The binary vector p2GWF7.0, harboring the double cauliflower mosaic virus 35S (CaMV35S)-ADH-green fluorescent protein (GFP), was introduced into freshly obtained *Populus* protoplasts by polyethylene glycol (PEG) and then incubated for 20 h at 23 °C before being observed and recorded. The GFP fluorescence signals were imaged using an OLYMPUS BX35 microscope (Olympus, Tokyo, Japan).

### 2.7. Populus Transformation and Waterlogging Treatment

The overexpressed binary vector PH35GS with *ThADH1* and *ThADH4* under the control of the CaMV35S promoter was transformed into *Agrobacterium tumefaciens* strain GV3101. The transformed *A. tumefaciens* strain was used to transform hybrid poplar as previously described [20]. After transformation, the non-transgenic plants and transgenic plants were grown on MS agar medium under 16/8 h of day/night at temperature of 25 °C for 30 days, Kanamycin was used to select for the transformation-positive plants and qPCR was performed to verify the putative transformants (Table 1). Before of the waterlogging treatment, 12 plants (4 non-transgenic *Populus*, 4 *ThADH1*-overexpressed transgenic *Populus* and 4 *ThADH4*-overexpressed transgenic *Populus*) were selected for the growth parameters analysis. The plant height was measured with ruler and then the plants were dried at 80 °C until constant weight to derive their dry weights, ME104E electronic balance (METTLER, Zurich, Switzerland) was used to measure biomass. Data of growth parameters were analyzed with one-way analysis of variance (ANOVA) followed by Duncan’s multiple range test at *p* < 0.05 and the expression profiles of *ThADH1* and *ThADH4* in non-transgenic and transgenic *Populus* were detected by qPCR. Moreover, 8 non-transgenic *Populus*, 12 *ThADH1*-overexpressed transgenic *Populus* (line 1–3), 12 *ThADH4*-overexpressed transgenic *Populus* (line 1–3), a total of 32 plants were selected for waterlogging experiment with tissue culture bottles (15 cm × 8 cm each). After 20 days, the waterlogging stress was removed to obtain phenotype observations, measure the biomass and plant height.

## 3. Results

### 3.1. Isolation and Characterization of ADH Genes from T. hybrid ‘Zhongshanshan 406’

Through the previous annotated transcriptome sequences, the full-length cDNAs of the two *ADH* genes, namely *ThADH1* and *ThADH4*, were cloned from the cDNA of *T*. hybrid ‘Zhongshanshan 406’, using the RACE technique. *ThADH1* (Genbank ID: AWL83216) proved to be highly homologous to gymnosperms *Pinus banksiana PbADH* (83.06%), whereas the homology of the amino acid sequence with that of *A. thaliana AtADH1* was 76.86%. The second *T*. hybrid ‘Zhongshanshan 406’ *ADH* gene, *ThADH4* (Genbank ID:AWL83217), shared 71% identity at the amino acid level with relict plant *Amborella trichopoda AmADH4* (Genbank ID: XP 011620525.2). Sequence analysis demonstrated that the cDNAs of *ThADH1* and *ThADH4* encoded proteins of 381 and 403 amino acids (Supplementary Data 1), respectively, with predicted molecular weights (MW) of 41.03 and 43.7 kDa, as well as isoelectric points (pI) of 6.23 and 5.91, respectively. Protein subcellular localization predictions, using transient expression assays, indicated that the two ThADH proteins were both located in the cytoplasm, whereas ThADH4 protein was also located in the nucleus. The ThADH1 and ThADH4 proteins contained 15.22% and 16.13% α helices (Hh), respectively, a 32.55% and 29.53% of extended strands (Ee), respectively and 52.23% and 54.34% of random coils (Cc), respectively. Interestingly, there was no zero β turns (Tt) in ThADH1 and ThADH4 proteins (Table 2).

### 3.2. Multiple Alignment and Phylogenetic Analysis of ThADH1 and ThADH4

**Figure 1:**
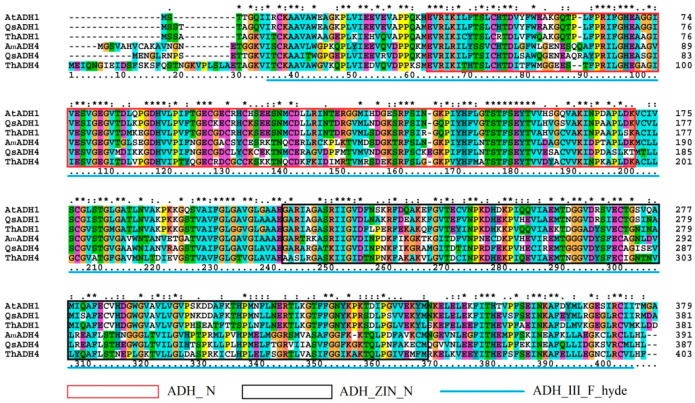
Alignment of the amino acid sequences of ThADH1, ThADH4 with *Arabidopsis thaliana* AtADH1, *Quercus suber* QsADH1, QsADH4 and *Amborella trichopoda* AmADH4. The highly conserved regions of the ADH proteins are shown by the red, black boxes and blue line, which represent the following conserved domains: ADH_ N domain, ADH_ZIN_N domain and ADH_III_F_hyde domain.

Multiple alignments were carried out to show the identity of the protein sequences between ThADH1, ThADH4 and other known ADH proteins AtADH1, *Quercus suber* QsADH1 (GenBank ID: XP_023885013.1), AmADH4 and QsADH4 (GenBank ID: XP_023893720.1). The result showed that both ThADH proteins contained a large ADH_III_F_hyde domain, indicating that ThADH1 and ThADH4 were members of the plant-specific class III alcohol dehydrogenase protein family, with a conserved ADH_ N domain located in the N-terminal and an ADH_ZIN_N domain located in the C-terminal of the ThADH1 and ThADH4 proteins. Although the amino acid sequences exhibited slight differences, the genes exhibited high similarities with one another in the ADH domain (Figure 1).

**Figure 2:**
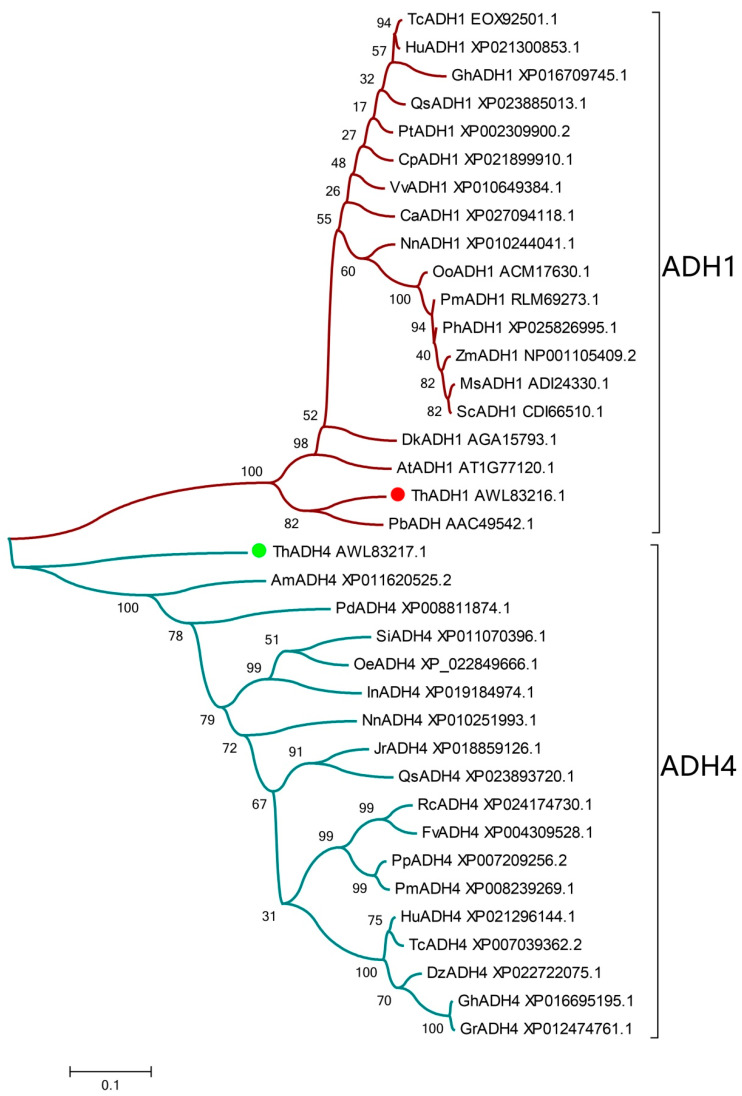
Phylogenetic analysis of the proteins of the *ADH* gene family. The phylogenetic tree was constructed using MEGA 7.0 with the maximum likelihood method using 1000 replicate bootstrap tests. Numbers near to the nodes indicate bootstrap values obtained from 1000 replications. The 35 proteins were clustered into two distinct groups: ADH1 and ADH4, as indicated. At, *Arabidopsis thaliana*; Os, *Oryza sativa*; Pb, *Pinus banksiana*; Pt, *Populus trichocarpa*; Hu, *Herrania umbratica*; Tc, *Theobroma cacao*; Vv, *Vitis vinifera*; Pa, *Prunus avium*; Qs, *Quercus suber*; Pm, *Panicum miliaceum*; Zm, *Zea mays*; Dl, *Dimocarpus longan*; Rc, *Rosa chinensis*; Gh, *Gossypium hirsutum*; Ca, *Coffea arabica*; Sh, *Saccharum* hybrid cultivar; Ms, *Miscanthus sinensis*; Nn, *Nelumbo nucifera*; Dk, *Diospyros kaki*; Oo, *Oryza officinalis*; Am, *Amborella trichopoda*; Nn, *Nelumbo nucifera*; Dz, *Durio zibethinus*; Gr, *Gossypium raimondii*; Rc, *Rosa chinensis*; Jr, *Juglans regia*; Oe, *Olea europaea* var. *sylvestris*; Pm, *Prunus mume*; Pp, *Prunus persica*; Pd, *Phoenix dactylifera*; In, *Ipomoea nil*; Se, *Sesamum indicum*; Fv, *Fragaria vesca* subsp. *vesca*; Zm, *Zea mays*; Cp, *Carica papaya*; Ph, *Panicum hallii*; Sc, *Saccharum* hybrid cultivar; Si, *Sesamum indicum*.

To further understand the evolutionary relationships between the ThADH proteins and the ADH proteins of other species. The amino acid sequences of ThADH1, ThADH4 and 35 ADH proteins from other species were used to construct a phylogenetic tree by MEGA7 software. Phylogenetic analysis, based on multiple sequence alignments, revealed that all these proteins could be divided into two distinct groups: ThADH1, closely related to AtADH1 and other ADH1 proteins, was positioned in the ADH1 clade, whereas ThADH4 belonged to the other group, including AmADH4, *Phoenix dactylifera* PdADH4 (GenBank ID: XP008811874.1), *Sesamum indicum* SiADH4 (GenBank ID: XP011070396.1) and so on, were located in the ADH4 clade (Figure 2).

### 3.3. Expression Patterns of ThADH1 and ThADH4 Genes

**Figure 3:**
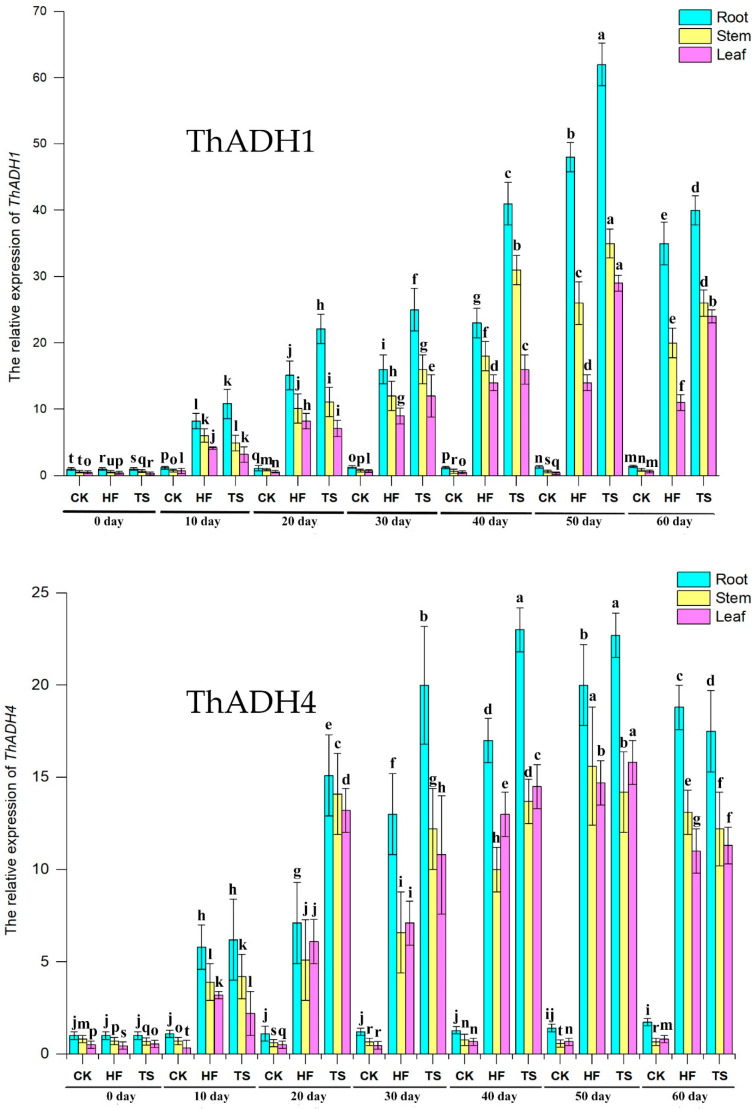
Temporal expression patterns of *ThADH1* and *ThADH4* by qPCR. CK, HF and TS represent non-flooding, half-flooding and total submergence. The gene expression level in the root at 0 day was set to 1. *ThADH1* and *ThADH4* genes expression in root, stem and leaf of *T*. hybrid ‘Zhongshanshan 406’at different times (from 0 day to 60 day). For qPCR, the *APRT* gene was used as the internal control and the relative transcript levels were calculated using the comparative delta-Ct method. All the qPCR data are shown as the mean ± standard deviation (error bar) of three biological replicates. Means with different letters are significantly different at *p* < 0.05 as determined by one-way ANOVA with Duncan’s multiple range tests. The same below.

To analyze the expression of *ThADH1* and *ThADH4*, the two *ThADH* genes were expressed in the three main organs (stems, leaves and roots) of entire *T*. hybrid ‘Zhongshanshan 406’ plants at three levels of treatments and seven time points, analyzed by qPCR (Supplementary Data 2). The mRNA expression patterns in response to waterlogging were similar, with an apparent increase–decrease trend. Gene expression in roots was much higher than that in stems and leaves under half-flooding (HF), total submergence (TS) and non-flooding (CK) conditions. The expression levels of the two genes exhibited a continuous rise in expression from 0day to 50day of the TS and HF treatments, in both roots or stems, the greatest accumulation of both *ThADH1* and *ThADH4* transcripts was observed on the 50th day, from which point gene expression began to decline (Figure 3).

### 3.4. Subcellular Localization of ThADH1 and ThADH4 Proteins

**Figure 4:**
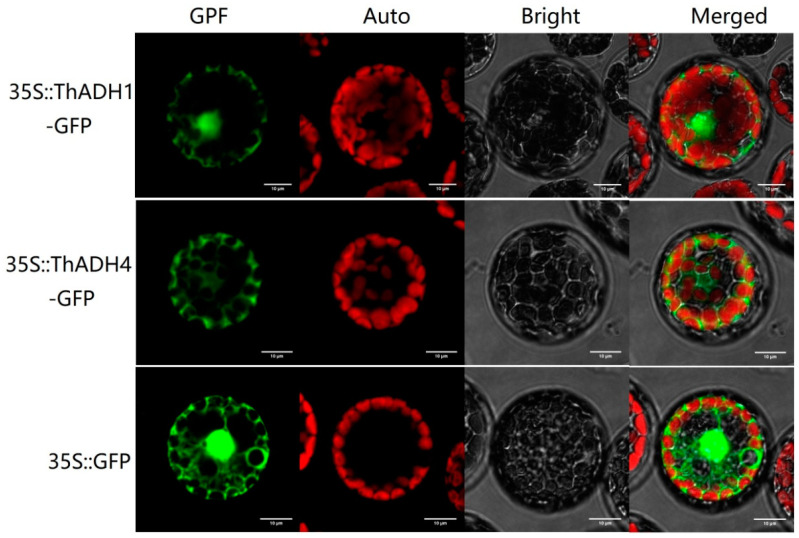
Subcellular localization of ThADH1 and ThADH4 in poplar protoplasts. Green fluorescent protein (GFP), chlorophyll autofluorescence (Auto), bright and merged images are shown. Scale bar = 10 µm. The 35::GFP fusion protein was used as the positive protein control.

In order to probe the subcellular localizations of the ThADH proteins, under the control of 35S CaMV promoter, the GFP was fused to the C-terminus of the ADH proteins and the chimeral genes introduced into hybrid poplar (*Populus davidiana* × *Populus bolleana*) protoplasts for expression in a transient gene expression assay. As a positive control, we detected 35S::GFP fusion protein signals in the nucleus, cytoplasm and cytomembrane of *Populus* protoplasts and the fluorescent signal of ThADH1 was detected within the nucleus and the cytoplasm, whereas no nuclear ThADH4 protein fluorescent signal could be found in the nucleus, which was located only in the cytoplasm (Figure 4). These results implied that ThADH1 and ThADH4 may have different biological functions in plants.

### 3.5. Heterologous Overexpression of ThADH 1 and ThADH 4 in Populus and Comparison of Waterlogging Tolerance in Non-Transgenic and Transgenic Populus

**Figure 5:**
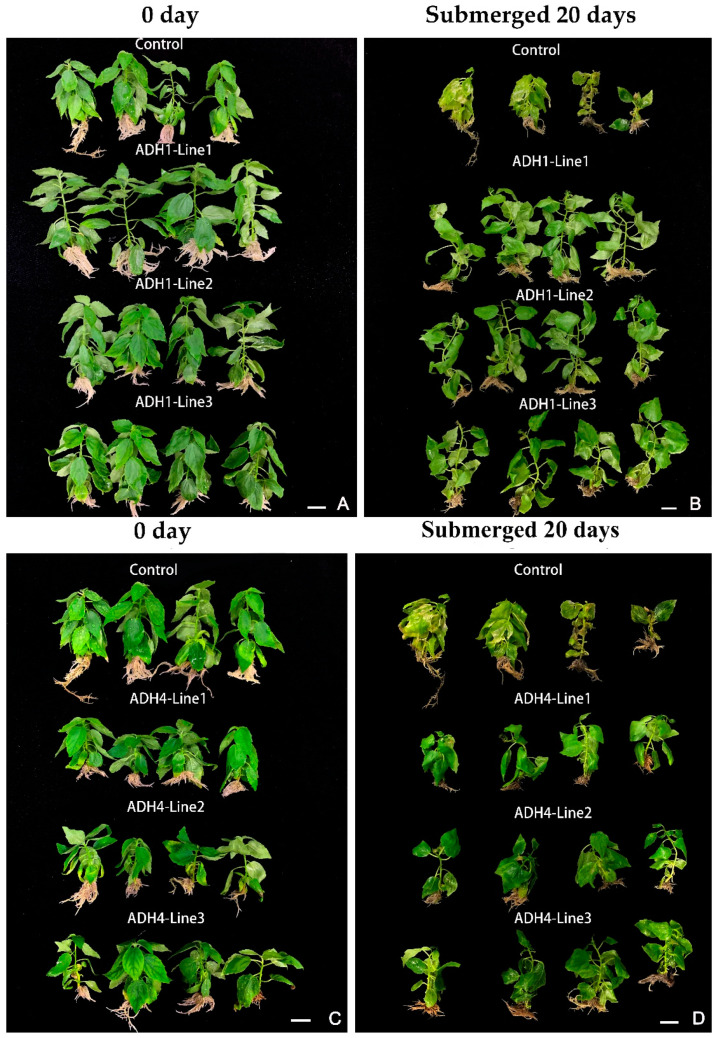
Overexpression of *ThADH1* or *ThADH4* conferred enhanced waterlogging tolerance in plants. (**A**,**C**) Phenotypic changes of transgenic *Populus* after 30 days of growth ADH1/ADH4 Lines 1–3 and non-transgenic (Control), respectively. (**B**,**D**) 30 days WT and transgenic *Populus* were photographed at the conclusion of submersion in deionized water for an additional 20 days. Bar scale = 1.0 cm.

To reveal the biological functions of *ThADH1* and *ThADH4*, we overexpressed *ThADH1* and *ThADH4* in *Populus*. Phenotypic observation demonstrated that the growth rates of the *ThADH1*- and *ThADH4*-overexpressed transgenic plants were significantly different from that of non-transgenic plants. After 30 days of growth, overexpression of the *ThADH1* accelerated the growth rate of the transgenic *Populus* but the growth rate of *ThADH4*-overexpressed transgenic *Populus* was lower than that of the control (Figure 5A,C). In particular, compared with non-transgenic *Populus*, the biomass and plant height of *ThADH1*-overexpressed transgenic *Populus* were higher by 15.6% and 49.4%, respectively (Table 3). Meanwhile, the petioles and stems of the *ThADH4*-overexpressed transgenic *Populus* were significantly shorter than that of non-transgenic and *ThADH1* transgenic *Populus*, resulting in a dwarfed phenotype. For this reason, the biomass and plant height of non-transgenic *Populus* was 50.4% and 51.2% higher than that of the *ThADH4* transgenic *Populus* (Table 3). The expression level of the *ThADH1* gene in the transgenic *Populus ThADH1* Lines 1–3 was 531–580 times higher than that in non-transgenic *Populus*, whereas the expression level of the *ThADH4* gene in the transgenic *Populus ThADH4* Lines 1–3 was 1331–1950 times higher than that in the non-transgenic *Populus*. Interestingly, *ThADH4* gene was detected in *ThADH1*-overexpressed transgenic *Populus* and *ThADH1* gene was also detected in *ThADH4*-overexpressed transgenic *Populus*, although their expression levels were relatively low (Supplementary PIC 4).

In order to further explore the waterlogging tolerance of *ThADH1* and *ThADH4*, we completely flooded 30-day-old *ThADH1*- and *ThADH4*-overexpressed transgenic plants for 20 days and the non-transgenic *Populus* was used as a control (Supplementary PIC5). Compared with *ThADH*1- and *ThADH4*-overexpressed transgenic *Populus*, the morphology of the non-transgenic *Populus* has changed significantly and its leaves and segments appeared to turn yellow or transparent, even rotted (Supplementary PIC6). However, the growth of *ThADH1*- and *ThADH4*-overexpressed transgenic plants was slightly affected at the terminal buds (Figure 5B,D) and some *ThADH1* transgenic *Populus* grew new adventitious roots under waterlogging stress (Supplementary PIC7). Furthermore, compared with non-flooding (at 0 day of flooding stress) plants, the biomass and plant height of *ThADH1*- and *ThADH4*-overexpressed transgenic *Populus* were significantly enhanced (13% and 4.6%, 10.8% and 15.2%, respectively) after submerged treatment for 20 days. However, non-elongating height and decreased biomass of non-transgenic plants were observed (Table. 3). These results implied that overexpression of *ThADH1* and *ThADH4* conferred tolerance to submergence and hypoxia stresses, albeit via different pathways and that they were the positive regulatory factors of waterlogging tolerance of *T*. hybrid ‘Zhongshanshan 406’.

## 4. Discussion

The plant *ADH* gene family is a small polygenic family, *A. thaliana* has only one *ADH* gene [21], whereas rice (*Oryza sativa*) and maize (*Z. mays*) have three copies of *ADH* genes each [22], whereas soybean (*Glycine max*) has six copies of *ADH* genes, five of which are induced to express under waterlogging stress [23]. At present, the study of *ADHs* is mainly concentrated on herbaceous plants, with few studies in woody plants. In this study, we described the identification and characterization of two *T*. hybrid ‘Zhongshanshan 406’ *ADH* genes, namely *ThADH1* and *ThADH4*. According to the results of National Center for Biotechnology Information (NCBI) homology alignment, *ThADH1* and *ThADH4* showed high similarities with respect to the amino acid sequences of ADH proteins from other plants. *ThADH1* shared 76.86% identity at the amino acid level with *A. thaliana AtADH1*, while *ThADH4* shared 71% identity at the amino acid level with *Amborella trichopoda AmADH4*, indicating that *ThADH1* and *ThADH4* are relatively highly conserved and this conservatism may be related to some specific functions. ThADHs are Zn-binding enzymes with two conserved domains: one ADH_N and one ADH_zinc_N domain. Both ThADHs have a large conserved DNA-binding domain (adh_III_F_hyde domain); almost all plant ADH proteins have these conserved domains, which may explain the reason for the highly conserved nature of plant alcohol dehydrogenases [24,25].

Recent studies have shown that most ADH proteins are localized in the cytoplasm. In upland cotton (*Gossypium hirsutum*), all 28 GhADHs were shown to be located in the cytoplasm, with some of the proteins also being located on the pericytoplasmic or extracellular membrane [26]. In this study, subcellular localization investigations showed that the ThADH4 protein was located only in the cytoplasm, whereas the ThADH1 protein was located not only in the cytoplasm but also in the nucleus. This result was consistent with our previous WoLF PSORT software predictions and may be related to the functions of the proteins and the biological processes involved. In *Arabidopsis*, hypoxia stress-related transcription factor RAP2.12 can specifically bind to ADH1 in the nucleus [27]. Therefore, we speculate that whether ThADH1 protein is located in the nucleus is related to the function of upstream transcription factors, which needs further study.

Under anaerobic conditions, alcoholic fermentation in higher plants is essential for supplying NAD^+^ to the glycolytic pathway, which is in charge of ATP synthesis and *ADH* genes are considered to play a key role in hypoxic stress response [28]. A total of 22 *TaADH* genes were identified from the wheat genome, three of which, namely *TaADH1*/*2*, *TaADH3* and *TaADH9*, played an important role in coping with waterlogging stress and which can be used as an effective basis on which to screen for waterlogging-tolerant wheat varieties [29]. Under conditions of hypoxia, the transcription levels of *ZmADH1* and *ZmADH2* in maize both increased rapidly and then decreased under anaerobic conditions [30]. In the current study, to further investigate the two isolated *ThADH* genes, we focused on their expression patterns under long-term waterlogging stress. The extent of up-regulation of *ThADH1* and *ThADH4* under waterlogging stress was consistent in the three organs tested, indicating that, although hypoxic stress was perceived by roots, the signal arrived shortly at the stems and leaves, inducing the expression of *ADH* genes in those organs. When plants were completely submerged in water, the expression of *ThADH1* and *ThADH4* were increased and the elevated expression pattern were continued until 50th day, when the expression levels of *ThADH1* and *ThADH4* in the roots reached to 63 and 23 times of 0 day, respectively. Research on rice and *Coix lacryma-jobi*, both grasses, have shown that both the *OsADH1* and *ClADH1* genes were sensitive to flooding stress, with the expression of these genes reaching the highest levels within six hours of short-term flood stress [31,32]. Results showed that there were some differences in expression response patterns of *ADH* genes between different plants under long-term and short-term waterlogging stress and that *ThADH1* and *ThADH4* could also be used as a marker of waterlogging stress. The sequences of *ThADH1* and *ThADH4* were similar to each other in terms of the ORF sequences; gene expression patterns also showed similar trends, although *ThADH1* was up-regulated to a much higher level than was *ThADH4* under waterlogging stress treatment. This result showed that, of the two genes, *ThADH1* may play the more important role in the response of *Taxodium* to hypoxic stress.

*ADH* is one of the most important candidate genes for genetic manipulation to provide waterlogging tolerance to plants via increasing adaptability to hypoxic responses. Maize and *Arabidopsis ADH*-null mutants showed lower tolerance to anaerobic conditions [33,34,35]. In rice, the inhibition of *ADH* resulted in a decrease in seed vigor under anoxic conditions, whereas overexpression of *ADH* promoted plant growth under waterlogging conditions [31,36]. However, the role of *ADH* during waterlogging-tolerance signaling has not been established in coniferous species. In the present study, we tested whether transgenic *Populus* carrying *ThADHs* exhibited increased waterlogging tolerance. Therefore, we overexpressed the two *ThADH* genes in hybrid poplars to identify their functions. Interestingly, transgenic plants *ThADH1* Lines 1–3 and *ThADH4* Lines 1–3 showed completely opposite phenotypes. Saika [18] demonstrated that a point mutation in the *OsADH1* gene is associated with inhibition of coleoptile elongation in rice under submergence conditions, indicating that *ADH* genes may affected the growth of plants. Sometimes, the dwarfing phenotype of plant may have a negative impact on the plant’s waterlogging tolerance but waterlogging experiments showed that overexpression of *ThADH1* or *ThADH4* genes enhanced the withstand flooding resistance of *Populus* (Supplementary Data 3). Compared with the control, the phenotype of *ThADH1-* or *ThADH4*-transgenic *Populus* was closer to the non-flooding state and they can grow normally under flooding stress. These results inferred that ThADH1 and ThADH4, as positive regulators, improve tolerance to waterlogging or hypoxia stresses of *T*. hybrid ‘Zhongshanshan 406’ through different signal pathways.

Taken together, these results strongly indicated that each of *ThADH1* and *ThADH4* plays an essential role in tolerance to waterlogging stress in *T*. hybrid ‘Zhongshanshan 406’. Further study is necessary to explore the response mechanism of *ThADH1* and *ThADH4* to waterlogging stress, to identify the association between them and other genes, such as *PDC* (*Pyruvate decarboxylase*) and *ALDH* (*Acetaldehyde dehydrogenase*in) the glycolysis pathway, to find their upstream and downstream genes and to explore whether the two genes function singly or together.

## 5. Conclusions

In this study, we isolated and characterized two *ThADHs* genes from *T*. hybrid ‘Zhongshanshan 406’. The expression levels of the two genes in roots, stems and leaves exhibited trends of continuous rise from 0 to 50 day under the half-flooding and total submergence treatments. Furthermore, we demonstrated that overexpression of *ThADH1* and *ThADH4* in *Populus* could significantly increase waterlogging tolerance of plant, inferring that *ThADH1* and *ThADH4* may play essential parts in tolerance to waterlogging stress in *T*. hybrid ‘Zhongshanshan 406’. Altogether, our findings will be beneficial to enrich the understanding of molecular mechanism of flooding tolerance in *T*. ‘Zhongshanshan’ and provide a theoretical basis for the germplasm innovation of *Taxodium*.

## Figures and Tables

**Table 1:** Primer sequences of the genes for rapid amplification of cDNA ends (RACE), open reading frame (ORF) and real-time quantitative polymerase chain reaction (qPCR).

Prime-ID	Forward PCR Primer (5’-3’)	Reverse PCR Primer (5’-3’)
*ThADH1*_3OUTER	AAAGAAAGCATTACAGAGGCGTG	AAATCACTAGTGGAACGACGGTA
*ThADH1*_3INNER	CAGTACCAGTACCAAATACCGAGGGTTGA	CCTATAGTGAAATCACTAGTGGAGGATCCGCG
*ThADH1*_5OUTER	CTAATACGACTCACTATAGGGCAAGCAGTGGTAT	TTTTTCCTCCATTTGCTCGTTCTCAA
*ThADH1*_5INNER	CTAATACGACTCACTATAGGGC	TCTGATTTCCGCAGCAAACTTTC
*ThADH1*_ORF	ATGTCAAGCGCTACTGCAGG	ATCATCCAGTTTCATGACACATCT
*ThADH1*_qRT-PCR	AGCGCTACTGCAGGGAAGGT	TTGATCCTGACTTCCATTGC
*ThADH4*_3OUTER	TTCAAGAAGTTATAGCAGAGATGA	AAATCACTAGTGGAACGACGGTA
*ThADH4*_3INNER	GGCCAAAACACAATTGCCTGGAATTGTGGAG	CCTATAGTGAAATCACTAGTGGAGGATCCGCG
*ThADH4*_5OUTER	GAAACGTGCTTTCTTCCCCTCCCAT	GCTTTCTATTGTATTGGGCTTCGTCTT
*ThADH4*_5INNER	GGATCCACCTGAACATCTTCTATTACCAGA	ACCAGACAAAGTTATTGGGAGCAGAGG
*ThADH4*_ORF	ATGGAGATACAGAATGGAATA	GAAGTGAAGAACACATCTCA
*ThADH4*_qRT-PCR	CAAAGTCCCTCTGTCT	AATATGCGAGGAAACGTG

**Table 2:** Features of the *ThADH1* and *ThADH4* genes and their protein products.

Gene_ID	Full-Length cDNA (bp)	5′-UTR (bp)	3′-UTR (bp)	ORF (bp)	Predicted Peptide			Secondary Structure Prediction			
					MW (kDa)	pI	GRAVY	Hh (%)	Ee (%)	Tt (%)	Cc (%)
*ThADH1*	1518	89	283	1146	41.03	6.23	−0.024	15.22	32.55	0.00	52.23
*ThADH4*	1506	117	177	1212	43.7	5.91	0.064	16.13	29.53	0.00	54.34

UTR: untranslated region; ORF: open reading frame; MW: molecular weight; pI: isoelectric point; GRAVY: grand average of hydropathicity; Hh: α helices; Ee: extended strands; Tt: β turns; Cc: random coils.

**Table 3:** Growth parameters of transgenic *Populus* and Non-transgenic *Populus* at 0d and 20d of flooding stress.

		Biomass (g)	Height (cm)
**0 Day**	*ThADH1*-Transgenic *Populus*	0.5449 ± 0.015b	11.5 ± 0.08b
	*ThADH4*-Transgenic *Populus*	0.3133 ± 0.009f	5.275 ± 0.09e
	Non-transgenic *Populus*	0.4713 ± 0.004c	7.975 ± 0.17c
**Submerged 20 Days**	*ThADH1*-Transgenic *Populus*	0.6161 ± 0.011a	12.025 ± 0.22a
	*ThADH4*-Transgenic *Populus*	0.3471 ± 0.014e	6.075 ± 0.09d
	Non-transgenic *Populus*	0.4131 ± 0.004d	8.05 ± 0.13c

Data presented are means ± standard errors (*n* = 4). Means followed by different letters in same column are significantly different according to Duncan’s Multiple Range Test at 5% level.

## Supplementary Materials

The following are available online at https://www.mdpi.com/2073-4425/12/2/225/s1, Supplementary Data 1: The full-length cDNA and amino acid sequences of *ThADH1* and *ThADH4*. Supplementary Data 2: Gene expression of *ThADH1* and *ThADH4*. Supplementary Data 3: Growth parameters of transgenic *Populus* and Non-transgenic *Populus* at 0d and 20d of flooding. Supplementary PIC 1–3. Normal irrigation (CK), half-flooding (HF) and total submergence (TS) of *T*. hybrid ‘Zhongshanshan 406’. Supplementary PIC 4: Histogram of gene expression in transgenic plants. Supplementary PIC 5: The 30-day-old non-transgenic plants, *ThADH1*- and *ThADH4*-overexpressed transgenic plants were completely flooded in tissue culture bottles. Supplementary PIC 6: Phenotypic changes of leaves and stem segments of some non-transgenic plants and transgenic plants after submerged 20 days. Supplementary PIC 7: The enlarged images of non-transgenic and *ThADH1*-overexpressed transgenic plants after 20 days of waterlogging treatment.
