# Supplementary material for: Identification and Functional Analysis of ThADH1 and ThADH4 Genes Involved in Tolerance to Waterlogging Stress in Taxodium hybrid ‘Zhongshanshan 406’
Source: Genes (Basel). 2021 Feb 4;12(2):225. doi: 10.3390/genes12020225 (PMC7913975; doi:10.3390/genes12020225)
Supplement: Supplementary file 1 [file genes-12-00225-s001.zip › Supplementary/S_Data 1.docx]

>ThADH1 cDNA

GAAAAGCAGTGGTATCAACGCAGAGTACATGGGGAATTCATTTTTACACCAAGTCATTAGATTTGTGCAAAGGAGATCTAACAACAAAAATGTCAAGCGCTACTGCAGGGAAGGTTATTACATGCAAAGCTGCCGTGGCATGGGCAGCAGGGGAGCCCCTTAAGATAGAACATGTGCAAGTGGCACCTCCACAGGCAATGGAAGTCAGGATCAAAATCTGCTACACTTCCTTGTGTCGCACAGACCTTGTTTTCTGGCAAGCAAAGGGCCAAACTCCACTTTTTCCACGCATCTTTGGACATGAAGCAGCTGGAATAGTAGAAAGTGTGGGAGAAGGGGTAACTGATATGAAGGAGGGTGACCATGTACTGCCTGTATTTACAGGGGAATGTGGAGATTGCAGGCACTGTAAATCAGAAGAAAGCAATATGTGTGATCTGCTAAGGATTAACACAGACCGAGGGGTTATGCTTAGTGATGGCAAGTCGAGGTTCTCTATCAGGGGACAACCCATTTATCACTTCTTGGGGACATCCACATTCAGTGAATACACAGTTGCACACGTTGGCTGTGTTGCCAAAATAAATCCTGCAGCTCCTCTCAGCAAAGCCTGTATTCTGAGCTGCGGTGTCTCCACAGGGATGGGAGCTACATTGAATGTGGCCAAACCTAAGAAAGGTTCAACCGTGGCAATATTTGGTCTCGGAGGTGTTGGGCTTGGGGCTGCTGAAGGAGCAAGAATTGCAGGAGCTTCTCGTATTATAGGCATCGATTTTCTCCCTGAAAGATTTGAAAAAGCAAAACAGTTTGGTGTGACAGAATATATCAACCCAAAAGACCATAAGAAACCTGTTCAACAGGTAATAGCAGAAAAAACTGATGGAGGAGCAGACTACAGTGTGGAATGCACAGGAAATATTAATGCAATGATTCAGGCTTTTGAATGTGTTCATGATGGGTGGGGTGTGGCAGTATTAGTAGGAGTGCCTCATTCAGAGGCAACTTTCACAACCAGTCCTCTCAACTTCCTCAATGAGAAAACATTAAAAGGGACATTCTTTGGGAACTACAAGCCCAAGTCAGATCTGCCAGGCCTTGTGGAAAAGTATCTGAGTAAGGAGTTTGAGCTGGAGGAGTTCATTACTCATGAAGTCCCTTTTGCTGAGATCAACAAGGCGTTTGATTTAATGGTGAAAGGAGAGGGCCTGAGATGTGTCATGAAACTGGATGATTAGATGGTGAAGGAGATTTTAGTAGCGTGGAAGGCTTTGGCTGGCTCATGGATTGCAATCGTTGAAAGATAATGGAGGCTTTTACATATGTCTGTAAATTTAAATAAAACCAGTTAGGTTAATAAGGGTTTATTGTAAAGGATTAAATAAAAGCTGGTATGATTGGGTGCAGATATATTAGGATTTTTTTTTGGTGTGTGTTCTAAATTACTTCTGTCAAAAGTCAATAAAGAAATTTTTCAAAAATTTAGTCTAAAAAAAAAAAAAAAAAAAAAAAAAAAAAAAA

>ThADH1 ORF

ATGTCAAGCGCTACTGCAGGGAAGGTTATTACATGCAAAGCTGCCGTGGCATGGGCAGCAGGGGAGCCCCTTAAGATAGAACATGTGCAAGTGGCACCTCCACAGGCAATGGAAGTCAGGATCAAAATCTGCTACACTTCCTTGTGTCGCACAGACCTTGTTTTCTGGCAAGCAAAGGGCCAAACTCCACTTTTTCCACGCATCTTTGGACATGAAGCAGCTGGAATAGTAGAAAGTGTGGGAGAAGGGGTAACTGATATGAAGGAGGGTGACCATGTACTGCCTGTATTTACAGGGGAATGTGGAGATTGCAGGCACTGTAAATCAGAAGAAAGCAATATGTGTGATCTGCTAAGGATTAACACAGACCGAGGGGTTATGCTTAGTGATGGCAAGTCGAGGTTCTCTATCAGGGGACAACCCATTTATCACTTCTTGGGGACATCCACATTCAGTGAATACACAGTTGCACACGTTGGCTGTGTTGCCAAAATAAATCCTGCAGCTCCTCTCAGCAAAGCCTGTATTCTGAGCTGCGGTGTCTCCACAGGGATGGGAGCTACATTGAATGTGGCCAAACCTAAGAAAGGTTCAACCGTGGCAATATTTGGTCTCGGAGGTGTTGGGCTTGGGGCTGCTGAAGGAGCAAGAATTGCAGGAGCTTCTCGTATTATAGGCATCGATTTTCTCCCTGAAAGATTTGAAAAAGCAAAACAGTTTGGTGTGACAGAATATATCAACCCAAAAGACCATAAGAAACCTGTTCAACAGGTAATAGCAGAAAAAACTGATGGAGGAGCAGACTACAGTGTGGAATGCACAGGAAATATTAATGCAATGATTCAGGCTTTTGAATGTGTTCATGATGGGTGGGGTGTGGCAGTATTAGTAGGAGTGCCTCATTCAGAGGCAACTTTCACAACCAGTCCTCTCAACTTCCTCAATGAGAAAACATTAAAAGGGACATTCTTTGGGAACTACAAGCCCAAGTCAGATCTGCCAGGCCTTGTGGAAAAGTATCTGAGTAAGGAGTTTGAGCTGGAGGAGTTCATTACTCATGAAGTCCCTTTTGCTGAGATCAACAAGGCGTTTGATTTAATGGTGAAAGGAGAGGGCCTGAGATGTGTCATGAAACTGGATGAT

>ThADH1 PROTEIN

MSSATAGKVITCKAAVAWAAGEPLKIEHVQVAPPQAMEVRIKICYTSLCRTDLVFWQAKGQTPLFPRIFGHEAAGIVESVGEGVTDMKEGDHVLPVFTGECGDCRHCKSEESNMCDLLRINTDRGVMLSDGKSRFSIRGQPIYHFLGTSTFSEYTVAHVGCVAKINPAAPLSKACILSCGVSTGMGATLNVAKPKKGSTVAIFGLGGVGLGAAEGARIAGASRIIGIDFLPERFEKAKQFGVTEYINPKDHKKPVQQVIAEKTDGGADYSVECTGNINAMIQAFECVHDGWGVAVLVGVPHSEATFTTSPLNFLNEKTLKGTFFGNYKPKSDLPGLVEKYLSKEFELEEFITHEVPFAEINKAFDLMVKGEGLRCVMKLDD

> ThADH4 cDNA

GAAAAATGGGGGAAGGATTTTGAGGTTTAGCAATTAGAAGAGAACTGCAAATCTCAGGTCGAGAATTCTTTTTGACAGTGTTTTCTATCCTTCGATTTTTGCTCTGTTGTTTGGACAATGGAGATACAGAATGGAATAGAAATTGACTCTTTCAGTAAGAGTTTTCAGAGTACAAATGGCAAAGTCCCTCTGTCTCTTGCAGAAACTGCTGGTAAAGTCATCACTTGCAAAGCTGCAGTAGCATGGGGAGTGAAGCAACCTCTGGTAATAGAAGATGTTCAGGTGGATCCTCCAAAATCAATGGAAGTCCGCATTAAAATCACCCACACCTCTCTCTGCCACACCGATATTACATTCTGGATGGGAGGGGAAGAAAGCACGTTTCCTCGCATATTGGGCCATGAGGGTGCTGGCATAATAGAGAGCGTGGGTGAGGGCATAACAGATCTTGTGCCTGGAGATCACGTGATTCCAACATACCAAGGAGAGTGTAGAGATTGTGGGTGTTGCAAATCTAAGAAAACCAATCAGTGTGACAAGTTCAAAATTGATATCATGAGAACAGTCATGAGAAGTGATGAGAAGAGCAGGTTTTCTTTGGGTGGGAAGCCGGTGTACCATTTCATGGCCACATCTACATTCAGCGAGTATACAGTCGTGGATTATGCTTGTGTTGTCAAAATTAATCCTAAAGCTCCTTTAGACAAAGCCTGCTTGCTTGGCTGTGGGGTAGCCACTGGTTTTGGAGCTGTGATGAATTTAACAGATATAGAAGTTGGATCAACAGTGGCTGTCTTTGGCCTGGGTACTGTTGGCCTTGCAGTTGCAGAAGCCGCAAGTCTAAGAGGAGCCTCAAAGATAATAGGAATAGATACCAATCCAAATAAGTTCGCCAAAGCCAAAGTATTGGGGGTAACCGACTGCATCAATCCAAAAGACCATGAGAAGCCCATTCAAGAAGTTATAGCAGAGATGACAAATGGAGGCGTGGATTACAGCTTCGAATGCATTGGAAATACCAACGTATTATACCAGGCCTTTTTGTCTACTAATGAGCCATTGGGGAAAACGGTGTTATTGGGACTGGACGCTAGTCCTCGCAAGATATGCCTCCATCCCTTGGAATTATTTTCAGGGAGAACTTTAGTGGCCTCCATCTTTGGAGGGATCAAGGCCAAAACACAATTGCCTGGAATTGTGGAGATGTTTATGCGTAAGGAGTTGAAAGTTGAGGAATATATTACTCACGAGTTCTCATTTTCAGAAATCAACAAAGCATTTGAATTGTTGTTGGAAGGCAATTGTTTGAGATGTGTTCTTCACTTCTAGACAAATTTGAAGGAATGGAAGATCTATTTCTGAATGGCAGATAGAAAGACATTTAGGTAGTTGGCTAAAATTACTTCTAAAACATGTGATGGTCTTAAGAATGTCCTTGTGTTTATCAAAAAAGAAAAGAAAGAATGTCCTTATTTTTATCAAAAAAAAAAAAAAAAAAAAAAAAAA

>ThADH4 ORF

ATGGAGATACAGAATGGAATAGAAATTGACTCTTTCAGTAAGAGTTTTCAGAGTACAAATGGCAAAGTCCCTCTGTCTCTTGCAGAAACTGCTGGTAAAGTCATCACTTGCAAAGCTGCAGTAGCATGGGGAGTGAAGCAACCTCTGGTAATAGAAGATGTTCAGGTGGATCCTCCAAAATCAATGGAAGTCCGCATTAAAATCACCCACACCTCTCTCTGCCACACCGATATTACATTCTGGATGGGAGGGGAAGAAAGCACGTTTCCTCGCATATTGGGCCATGAGGGTGCTGGCATAATAGAGAGCGTGGGTGAGGGCATAACAGATCTTGTGCCTGGAGATCACGTGATTCCAACATACCAAGGAGAGTGTAGAGATTGTGGGTGTTGCAAATCTAAGAAAACCAATCAGTGTGACAAGTTCAAAATTGATATCATGAGAACAGTCATGAGAAGTGATGAGAAGAGCAGGTTTTCTTTGGGTGGGAAGCCGGTGTACCATTTCATGGCCACATCTACATTCAGCGAGTATACAGTCGTGGATTATGCTTGTGTTGTCAAAATTAATCCTAAAGCTCCTTTAGACAAAGCCTGCTTGCTTGGCTGTGGGGTAGCCACTGGTTTTGGAGCTGTGATGAATTTAACAGATATAGAAGTTGGATCAACAGTGGCTGTCTTTGGCCTGGGTACTGTTGGCCTTGCAGTTGCAGAAGCCGCAAGTCTAAGAGGAGCCTCAAAGATAATAGGAATAGATACCAATCCAAATAAGTTCGCCAAAGCCAAAGTATTGGGGGTAACCGACTGCATCAATCCAAAAGACCATGAGAAGCCCATTCAAGAAGTTATAGCAGAGATGACAAATGGAGGCGTGGATTACAGCTTCGAATGCATTGGAAATACCAACGTATTATACCAGGCCTTTTTGTCTACTAATGAGCCATTGGGGAAAACGGTGTTATTGGGACTGGACGCTAGTCCTCGCAAGATATGCCTCCATCCCTTGGAATTATTTTCAGGGAGAACTTTAGTGGCCTCCATCTTTGGAGGGATCAAGGCCAAAACACAATTGCCTGGAATTGTGGAGATGTTTATGCGTAAGGAGTTGAAAGTTGAGGAATATATTACTCACGAGTTCTCATTTTCAGAAATCAACAAAGCATTTGAATTGTTGTTGGAAGGCAATTGTTTGAGATGTGTTCTTCACTTCTAG

>ThADH4 PROTEIN

MEIQNGIEIDSFSKSFQSTNGKVPLSLAETAGKVITCKAAVAWGVKQPLVIEDVQVDPPKSMEVRIKITHTSLCHTDITFWMGGEESTFPRILGHEGAGIIESVGEGITDLVPGDHVIPTYQGECRDCGCCKSKKTNQCDKFKIDIMRTVMRSDEKSRFSLGGKPVYHFMATSTFSEYTVVDYACVVKINPKAPLDKACLLGCGVATGFGAVMNLTDIEVGSTVAVFGLGTVGLAVAEAASLRGASKIIGIDTNPNKFAKAKVLGVTDCINPKDHEKPIQEVIAEMTNGGVDYSFECIGNTNVLYQAFLSTNEPLGKTVLLGLDASPRKICLHPLELFSGRTLVASIFGGIKAKTQLPGIVEMFMRKELKVEEYITHEFSFSEINKAFELLLEGNCLRCVLHF
