# Supplementary figures and images for: Identification and Functional Analysis of ThADH1 and ThADH4 Genes Involved in Tolerance to Waterlogging Stress in Taxodium hybrid ‘Zhongshanshan 406’
Source: Genes (Basel). 2021 Feb 4;12(2):225. doi: 10.3390/genes12020225 (PMC7913975; doi:10.3390/genes12020225)

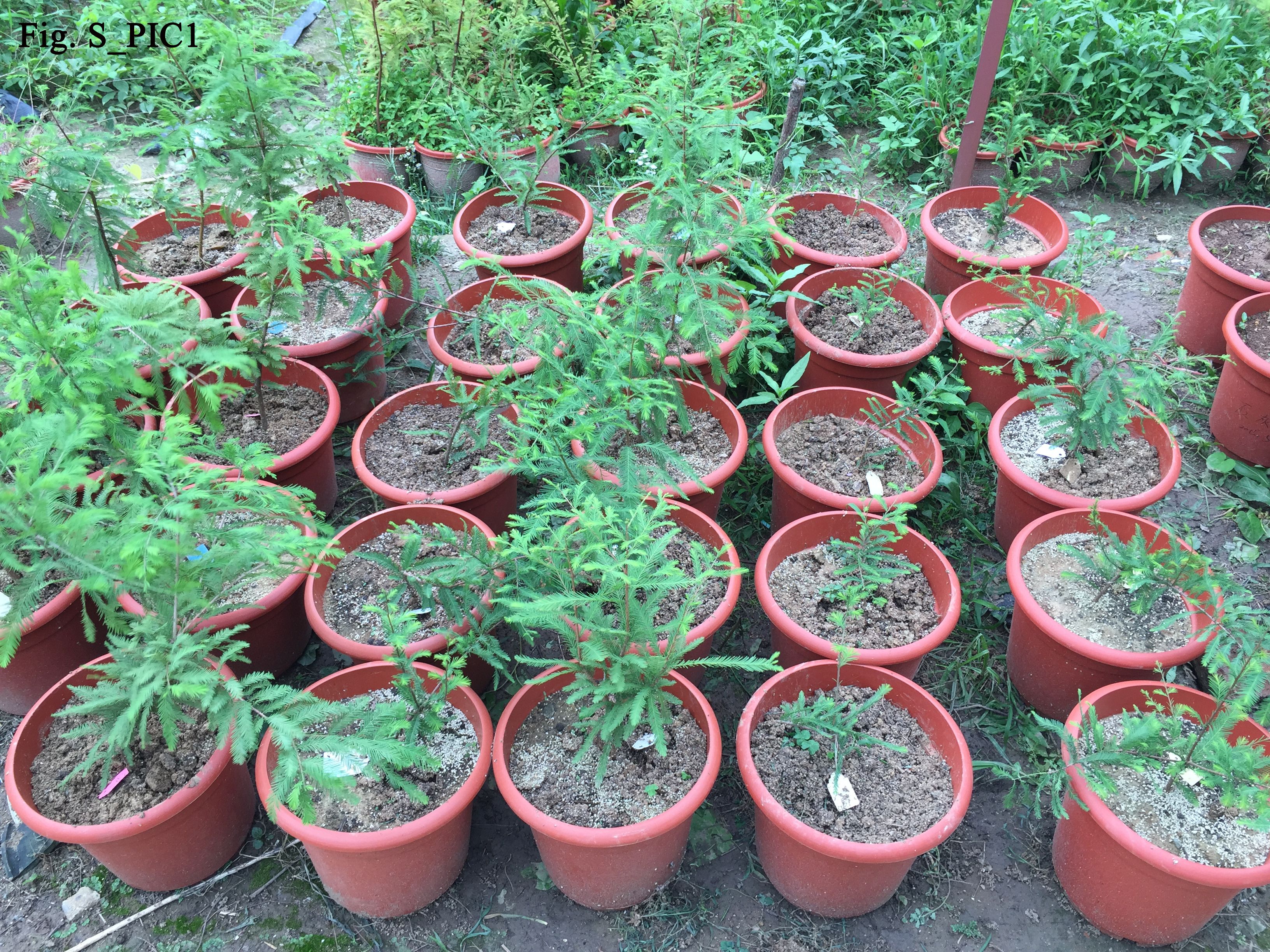

Supplement: Supplementary file 1 [file genes-12-00225-s001.zip › Supplementary/S_PIC 1.JPG]

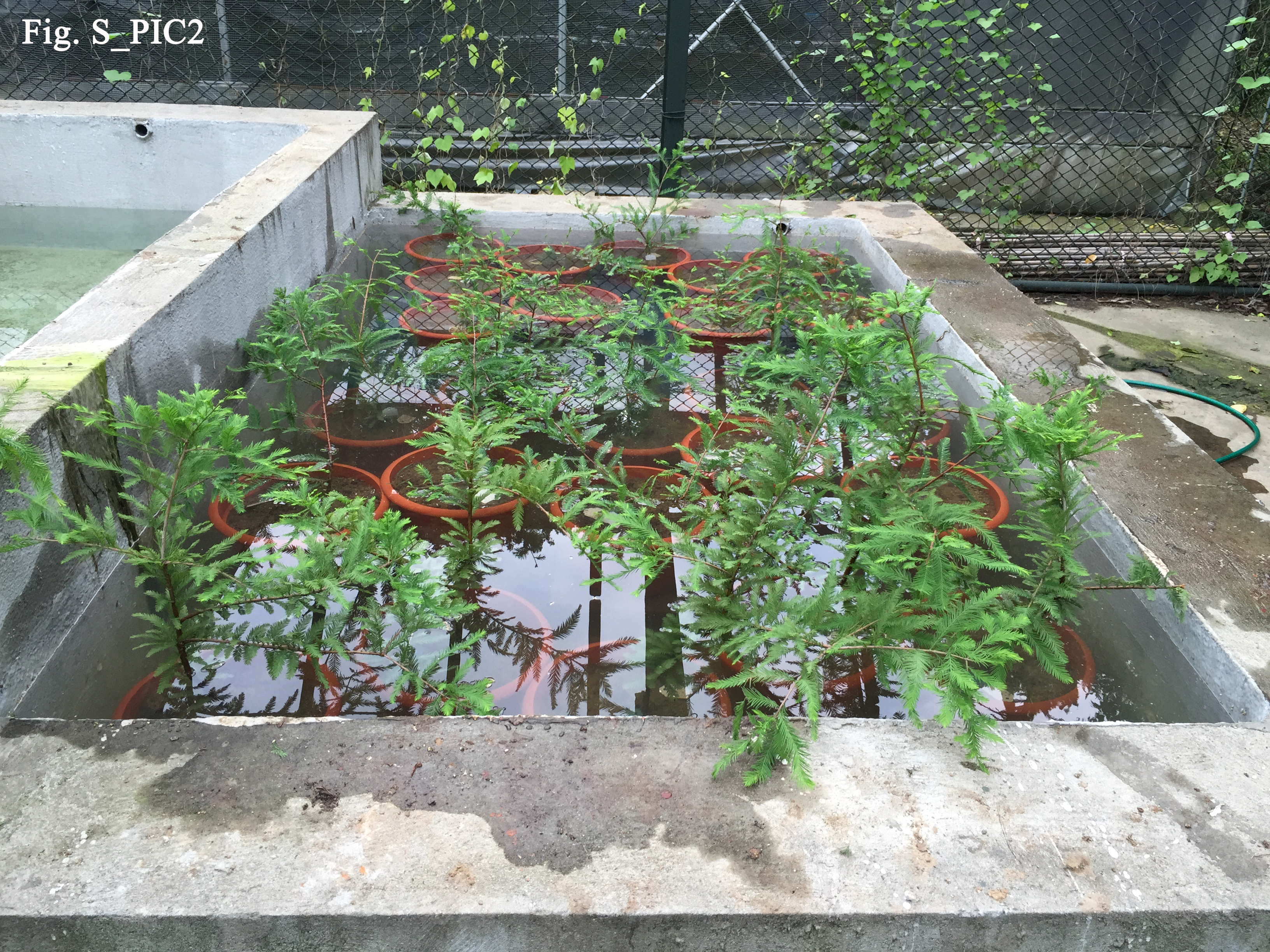

Supplement: Supplementary file 1 [file genes-12-00225-s001.zip › Supplementary/S_PIC 2.JPG]

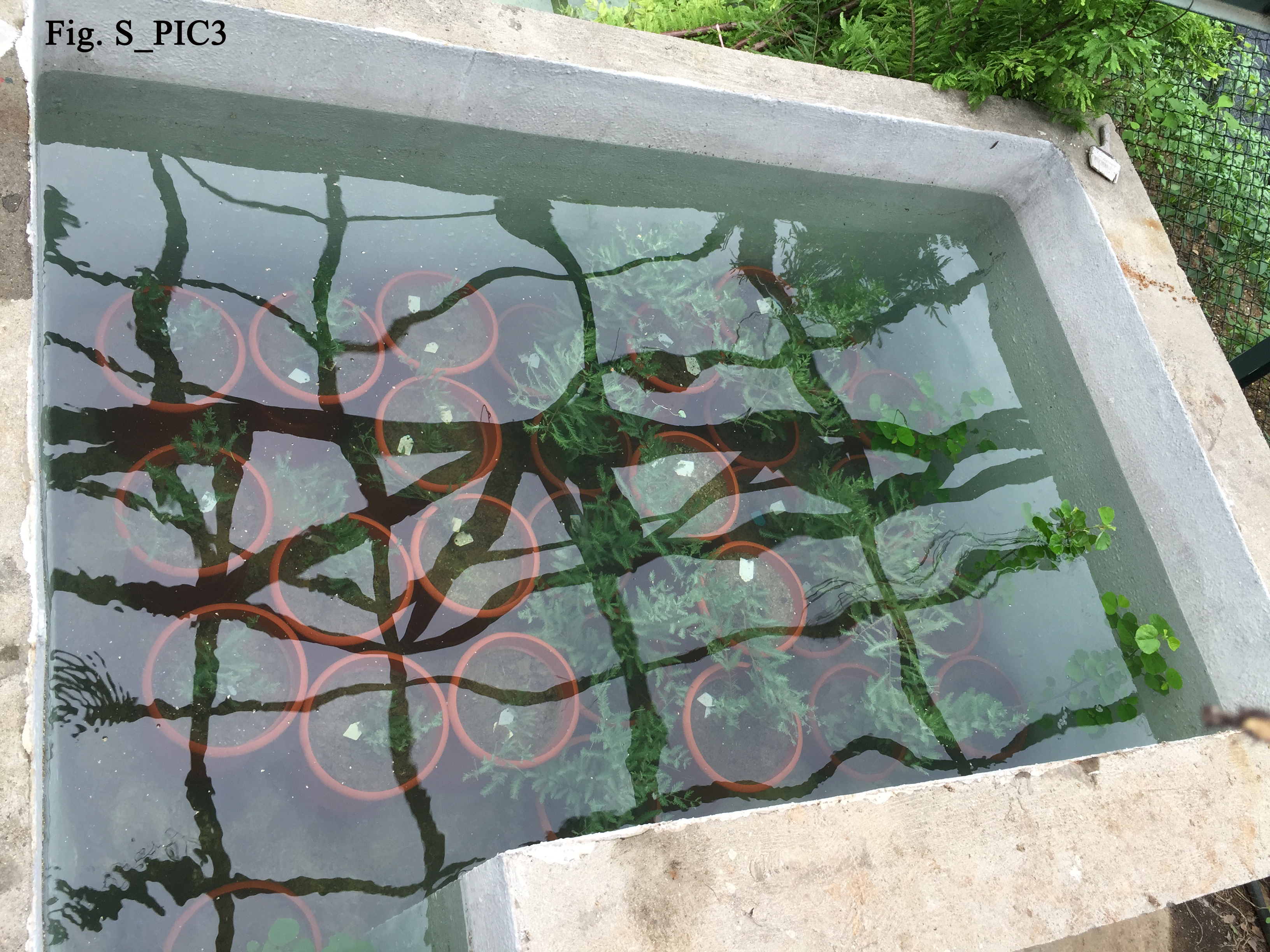

Supplement: Supplementary file 1 [file genes-12-00225-s001.zip › Supplementary/S_PIC 3.JPG]

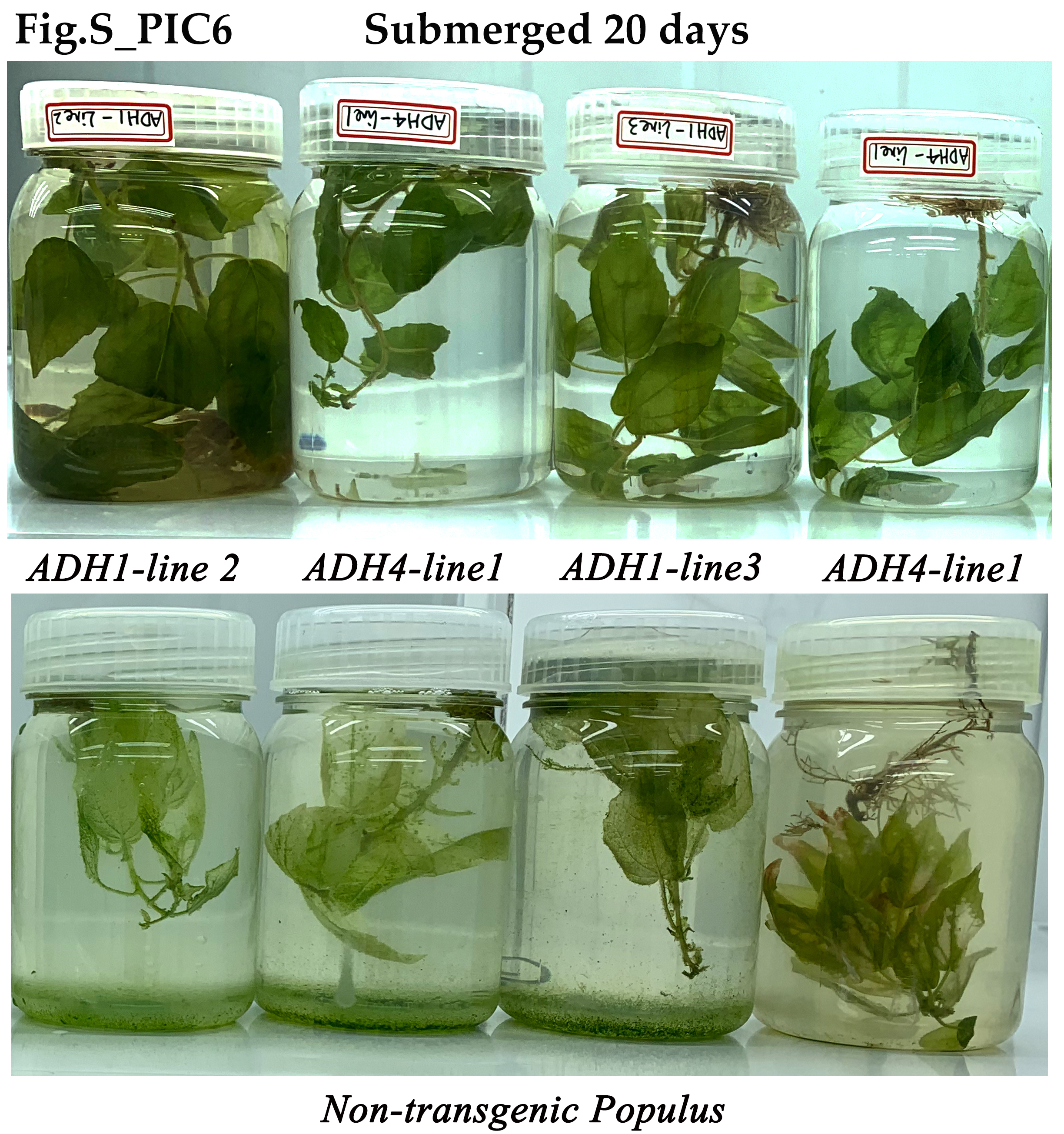

Supplement: Supplementary file 1 [file genes-12-00225-s001.zip › Supplementary/S_PIC 6.jpg]

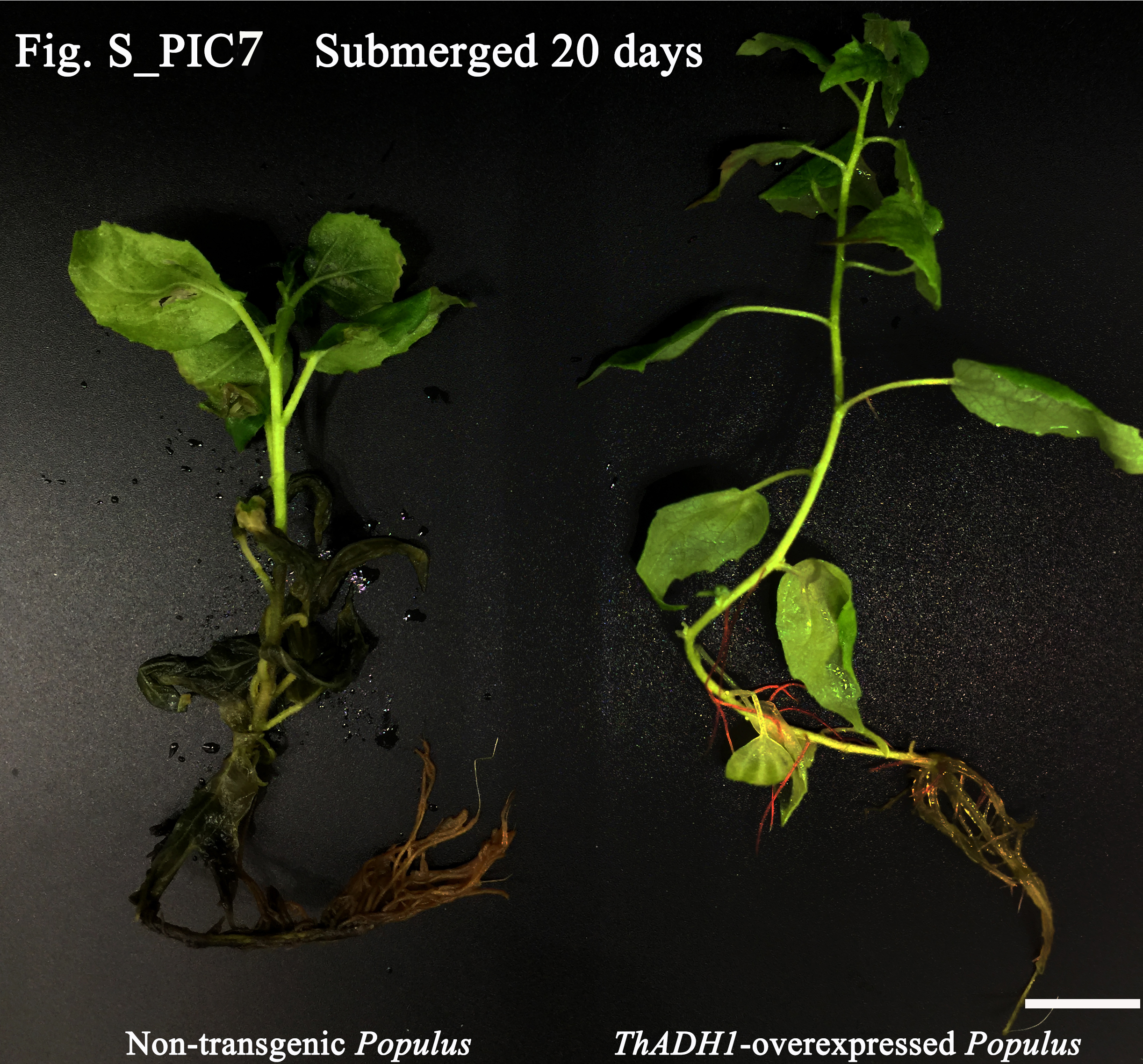

Supplement: Supplementary file 1 [file genes-12-00225-s001.zip › Supplementary/S_PIC 7.jpg]

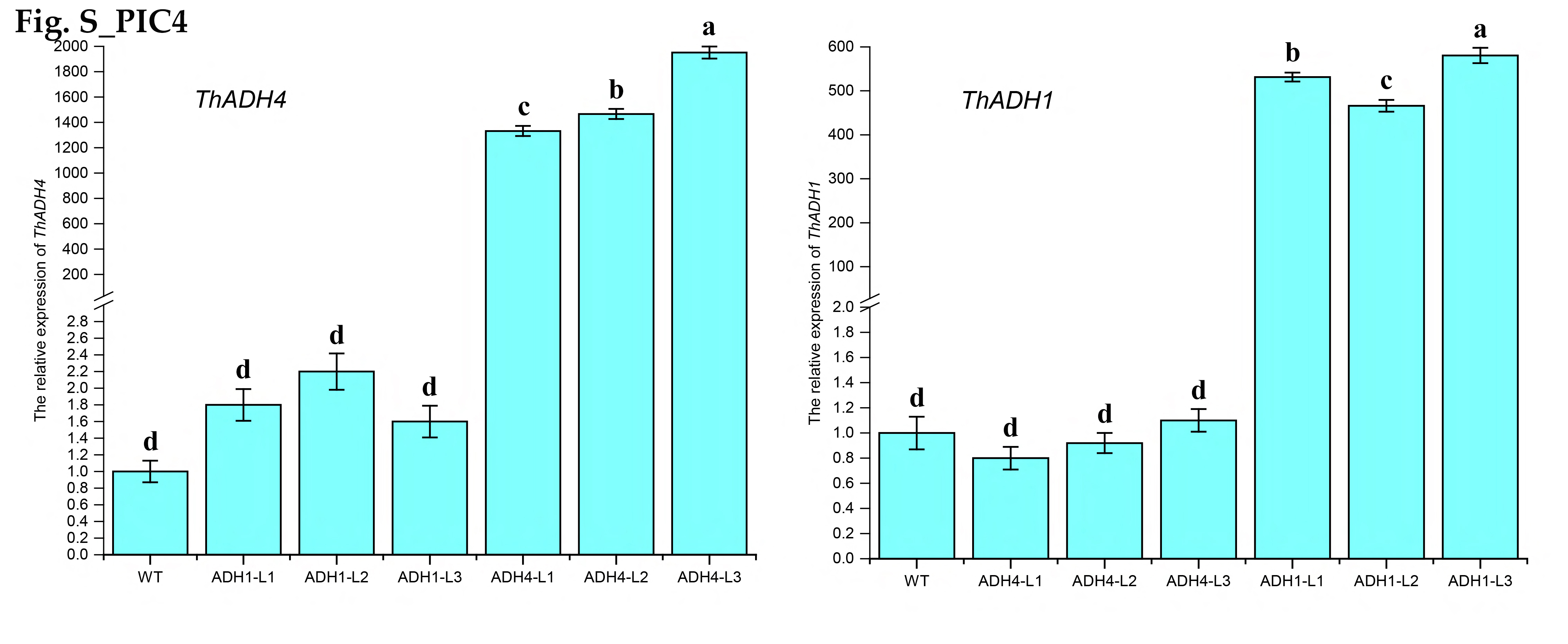

Supplement: Supplementary file 1 [file genes-12-00225-s001.zip › Supplementary/S_PIC4.jpg]

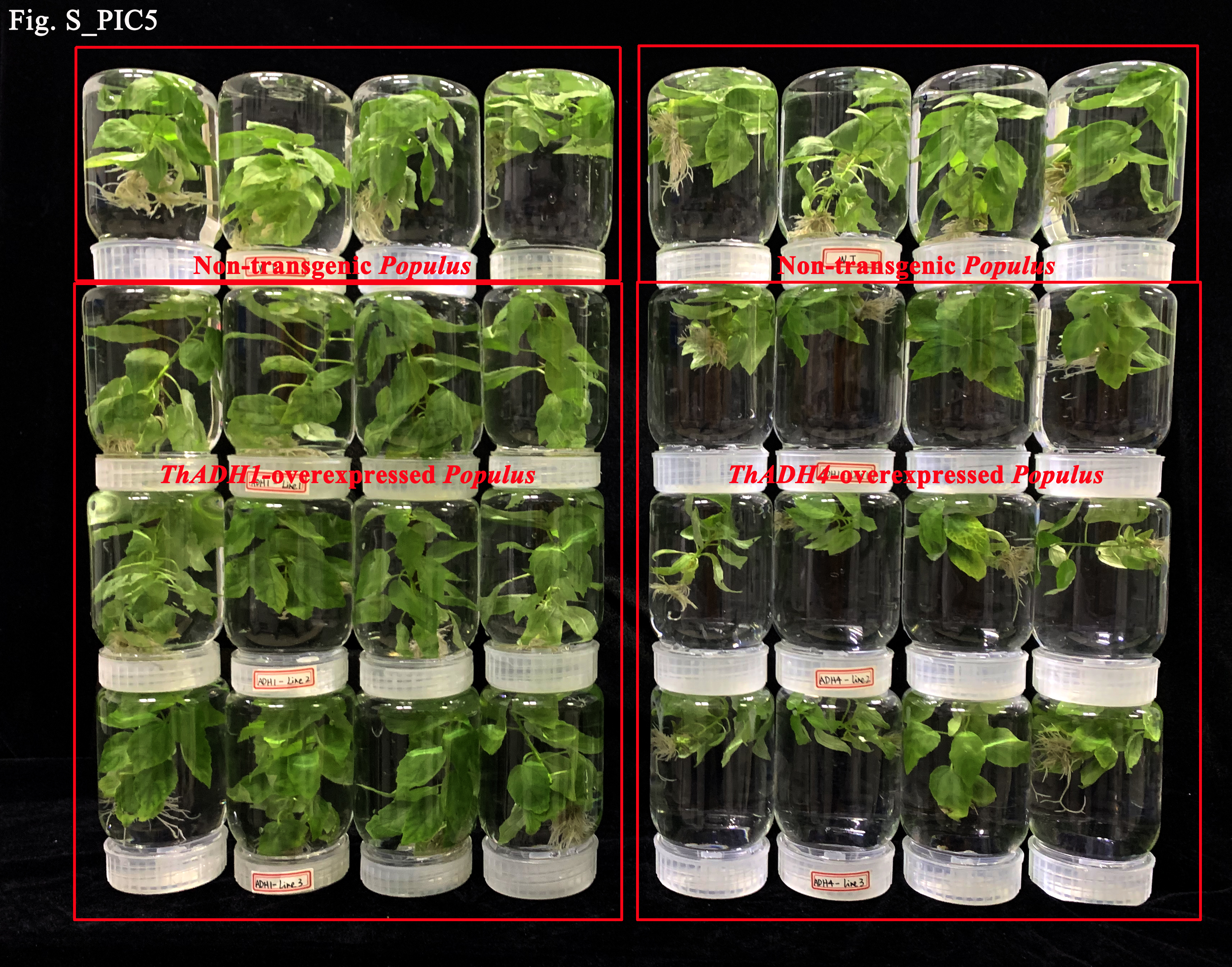

Supplement: Supplementary file 1 [file genes-12-00225-s001.zip › Supplementary/S_PIC5.jpg]
